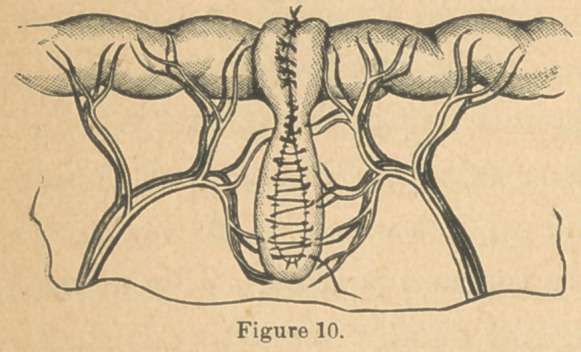# Gun-Shot Wounds of the Small Intestines

**Published:** 1884-07

**Authors:** Charles T. Parkes

**Affiliations:** Professor of Anatomy in Rush Medical College, Chicago, Ill.


### SELECTIONS.

GUN-SHOT WOUNDS OF THE SMALL INTESTINES. *By* CHARLES T. PARKES, M.D., *Professor of Anatomy in Rush Medical College, Chicago, Ill.*

[*Address of the Chairman of the Section on Surgery and Anatomy.*]

*Mr. President and Gentlemen of the American Medical Association:* The subject-matter of the remarks to be presented this morning was suggested to me by an article published in the *British Medical Journal* in 1882, from the pen of "that good man among men, and great man among doctors," J. Marion Sims.

The article in question was an appeal for operative interference in penetrating gun-shot wounds of the abdomen, in lieu of the "expectant treatment" so universally accepted and adopted by the profession, and which, in a few seemingly well authenticated instances, has led to recovery.

The appeal was uttered in behalf of the vast majority on the side of fatality attending these cases, and was based upon the deductions to be drawn from the recoveries following operations for diseases affecting the viscera of the abdomen and pelvis, during which the most terrible injuries have been inflicted upon the contents of these cavities—the peritonæum exposed for hours, as well as brought in contact with all kinds of foreign and usually irritating substances.

It is scarcely necessary for me to affirm in your presence the fact that, with few exceptions, the older writers and surgeons advocate the "expectant treatment" in the management of these injuries, while the younger writers and surgeons favor operations, pinning their faith upon the wonderfully favorable results attending the practice of Listerism, the purest of antiseptic surgical methods.

During the past few months I have instituted and carried out, with the valuable assistance of Mr. J. McDill and Drs. Anthony, Freer and Bolles, a series of experiments for the purpose of ascertaining the results to be obtained by immediate operations after these wounds, with the hope that the relation of the attending circumstances and events would be interesting as well as useful, by adding to the data now in our possession other data, from which may be determined more intelligently the course of action to be adopted when these cases come under our charge for treatment.

No attempt will be made to review the great question of penetrating gun-shot wounds of the abdomen, which would lead me beyond the scope of the paper. Nothing but a fair recital of the history of the experiments, with some application of the conclusions to be drawn therefrom, will be undertaken. With this intent in view, there will be presented to you the accompanying phenomena, the manner of treatment and results of thirty-seven intentional gun-shot wounds of the abdomen, confining my attention entirely to my own observations, and exhibiting to you such specimens as I have been able to preserve, taken from the animals; both of those which died, and of those which were sacrificed, after recovery, to obtain the specimen. Experiments of like nature have been made upon animals by very many surgeons, previous to the application of their convictions of the necessity of certain procedures to relieve disease or the effects of injury on the human body.

No preparation of the animals selected for experiment was made, either as to choice of physical condition or surrounding circumstances, except that they were anæsthetized previous to being hurt. The wounds were produced by the ordinary Smith and Wesson revolver of 22, 32, 38 and 44 caliber, and by the 22 caliber rifle. The shots were given at short range, so the damage done by the bullet fairly represents the injury met with, either in military or civil practice, as the results of shots from the firearms now in use.

At first, no attempt was made to give a definite direction to the course of the bullet, other than that it should perforate the abdominal cavity. The results soon confirmed the fact so well known, that the larger number of patients suffering from such wounds never come into the hands of the surgeon, their injuries proving rapidly fatal.

This ending, we can readily understand, must be a common one, when we bear in mind the construction and nature of the viscera contained in the cavity, especially their great vascularity, having vessels of immense size supplying them with, and carrying away from them, the blood necessary for their nutrition and the performance of their special functions; not to mention the main systemic artery and vein coursing through the cavity in a position rendering them readily liable to perforation, death following speedily.

It was also ascertained that a severe perforating and lacerated bullet wound of the viscera, such as of the kidneys, of the spleen, and of the pancreas, could not apparently be treated successfully in any other way than by an absolute removal of the injured organ; and notwithstanding the reported successful removal of almost every important organ of the abdomen by one surgeon or another, the conclusion was reached that some of these organs must be left *in situ*, in order that the functions of life may be carried on.

Hence we were compelled to exert such control over the course of the missile as to have it produce a wound of the nature of those likely to come, and actually coming, under the care of the surgeon; so that the injuries became those confined to perforations and injury of the intestinal tube, with occasionally the injury of some of the larger special organs.

It will not be amiss to recall to your minds, very briefly, some of the triumphs of abdominal surgery, and more especially to impress the fact that shot wounds of the cavity and contents present many questions of prime importance which are not met with in, and do not complicate, ordinary operations for disease or injury with any free, external wound.

The removal of the spleen for acute wounds nearly always results in recovery; so also one kidney has been removed successfully, either for disease or injury, often enough to place the operation of nephrectomy among the list of justifiable undertakings.

Again, wounds of the intestinal tube of all degrees of severity, up to complete division by the resection of portions of the entire caliber thereof, have been successfully treated by surgeons, as is proved by the experimental researches of Dr. Traverse, the eminent Prof. S. D. Gross, Dr. Bell, and others, and confirmed by the experience of many surgeons during operations upon the human being for diseases of these cavities. Still, in each of the examples mentioned, the circumstances were entirely different from what is found present in perforating gun-shot wounds of the abdomen. In the former, the peritoneal cavity was clear of blood and other extraneous substances; the prevention of their entrance entirely under the control of the operator. In the latter, blood in large amounts was always found present; and the peritonæum was smeared with the contents of the intestinal tube, necessitating prolonged efforts to secure a cavity clear of all hurtful substances. Of necessity, the latter cases would be least likely to escape the probabilities and dangers of subsequent inflammation of the serous membrane.

Primary resection of portions of the intestinal tube, or entire removal of separate organs, are operations comparatively easy of performance, and are not necessarily attended with any damage to or exposure of any other portions of the abdominal cavity, outside of the immediate proximity of the site of the operation.

Extravasation and hæmorrhage should be entirely prevented and controlled; and the peritoneal sac can be maintained perfectly clean during the time of, and after, all the procedures required by the operation.

After gun-shot wounds, besides the resection or removal of any special organ required, there is great shock, and prolonged manipulation is necessary to obtain a proper cleanliness.

The recital in detail of each experiment would be tiresome and occupy too much time, so that your attention will be called only to the more important facts and circumstances determined by them.

There will be published with the paper a somewhat extended account of each experiment, from which individual inferences may be drawn. In addition, a short *resume* of the entire work will be given further along.

First comes the question of hæmorrhage and damage to blood-vessels, as this is primarily the most common and certain cause of death, and demands the surgeon's first attention. In its excessive amount, occurring rapidly and suddenly, is to be found the explanation of the cases which are immediately fatal. This result will surely happen when the largest arterial trunks are severed by the bullet; further, its copiousness and persistency of flow, even when none but very small blood-vessels are divided, involve a matter of serious concern, if not a fatal issue, either from the amount of blood lost, or in predisposing to septic processes from blood decomposition.

There is a remarkable persistency in the flow of blood following the severance of vessels in the abdominal cavity, perhaps dependent upon the laxity of the tissues through which these vessels course, the absence of pressure from surrounding soft parts, and the lack of the peculiar influence of the atmosphere, either from its weight or clot-producing power.

When the abdomen is opened immediately after the transit of a bullet, its cavity is found to contain a large amount of blood, the quantity, of course, being in proportion to the size of the vessels wounded, but always a disproportionately large amount, no matter what their caliber; further, the flow is still going on from vessels of all sizes. There seems to be slight disposition to the formation of an obstructive clot in the mouths of the smaller ones, and slow retraction or contraction of the walls of the larger.

Bleeding stops only when the heart ceases to beat in a faint from excessive loss, or when the amount of blood is so large that by its bulk, and weight, and distension of the abdominal walls, it makes pressure sufficient to occlude the open vessels.

The conditions are very quickly altered after air is admitted through the abdominal section. Clots rapidly seal up the smallest vessels; the smaller arteries spurt less forcibly and soon cease beating; the larger ones contract and retract, just as occurs in the wounds of soft parts in other regions of the body. This is in accordance with, and corroborative of, the experience in hæmorrhages occurring in abdominal surgery in the human being. Few of us have failed to see cases like this: a patient dies suddenly, with all the symptoms of acute prostrating hæmorrhage; post-mortem examination shows the abdominal cavity filled with blood; the blood is carefully cleared away in the search for the source whence it came; and when this is found, it is a matter of astonishment that such a vast amount of blood could come from so small a vessel. Perhaps it is a small vein of the ovarian venous plexus, or a minute vessel in the thin-walled sac of an extra-uterine fœtation, or the partially closed vessels in the shrunken stump of a recently removed ovarian or other tumor, or some recently divided adhesions, all of them vessels which, in any other part of the body, would be no item of concern to the surgeon, or need any of his special care to prevent bleeding from them.

The lesson taught by these facts is of imperative importance in all operations upon these cavities; and even if mastered, loses nothing by reiteration. Excessive hæmorrhage being certainly the principal cause of speedy death in severe gun-shot wounds in this region of the body, where evidences of its presence are plainly exhibited, there can be no hope whatever of saving the lives of any of the wounded except by immediate abdominal section. This alone, by admitting air quickly, staunches the fast flowing current, and gives time for the application of the ordinary rules of surgery for the prevention of hæmorrhage.

In order to be safe from subsequent trouble, every divided blood-vessel must receive the surgeon's attention, occluding clots must be thoroughly sponged away, and in their stead must be placed the ligature or the sear of the actual cautery. If left without this restraint, and the abdominal opening be closed, the same conditions are restored as existed previous to the section; and as reaction comes on, bleeding will surely recur, and in large amount, leading to death from this cause alone, or furnishing a frequent source of septicæmia.

This fact again is corroborative of the experience of ovariotomists, the most successful being those who take the greatest pains to staunch all bleeding before closing the abdomen.

Following a resection of three or four inches of bowel and a ligation of two large subdivisions of the mesenteric artery wounded by the bullet, there occurred a mortification of several inches of the entire intestine above the site of resection. The mortified part corresponded with the distribution of the arteries wounded and ligated. This assuredly was an important fact to know, if at all likely to occur as the result of wounds of these arterial branches; even its accidental occurrence is a circumstance to be remembered. Its occurrence would surely add largely to the gravity of the cases in which it happened, probably necessitating a resection of a portion of the intestine corresponding to the area of distribution of the wounded vessel. The great freedom of anastamosis between the mesenteric arteries rather argues against their wounds being followed by any such hazardous result; still, the case recorded above required explanation. Two experiments were performed in order to determine whether destruction of the arteries alone was sufficient to lead to such mortification.

Both demonstrated that a closure of two or three of the largest subdivisions of the main mesenteric vessel was not in itself sufficient to produce death of the portion of intestine supplied by them. The experiments were as follows: an animal was anæsthetized, and the abdomen opened. A sufficient length of bowel was drawn through the opening to allow of the ligation of two large sets of vessels adjoining each, the ligatures including vein and artery. The parts were returned to the abdomen and the latter closed. At the end of thirty-six hours the wound was reopened. No very noticeable change was found in the intestine; pulsation had returned in the ligated vessels beyond the ligature. The external wound was again closed. The animal recovered in a few days so as to be as lively as ever.

A second animal was etherized, and a ventral section made. Three large vessels were ligated (veins and arteries), before their division into any branches. These three vessels lay parallel with each other. A ligature was also thrown around the anastomosing branch near the intestine which connected with a fourth larger vessel. There followed immediately very marked whitening of the bowel. The parts were returned and the wounds closed. The animal recovered promptly from the effects of the ether and the immediate effects of the operation.

It remained quite well for six days, when it grew ill. The wounds were reopened. Pulsation had returned beyond the ligature. There was no sloughing or mortification of the intestine. It was congested slightly and seemed paralyzed, and was of wider caliber opposite the distribution of the ligated vessels; this was the only change. There was a great deal of very offensive matter in the peritoneal sac, and notwithstanding the high grade of inflammation, there was no adhesion of intestinal folds except at one point. Here there was found a perforation of the intestine. Out of the opening there protruded a piece of wood which, upon being pulled out from the cavity of the intestine, was found to be four inches long, and connected with a large mass of twine. This had evidently been swallowed by the animal, and had gotten along safely enough until it reached the inactive portion of the tube corresponding to the seat of operation, where it was forced through the tube by the strong contractions behind it. Unfortunately, the animal was killed by the ether during the examination. Aside from this accident, the animal had a good chance of recovery.

The complication of a complete resection of the bowel, with a ligation of two or more vessels, is the only explanation to be given of the case where mortification occurred. The experiments prove that such result does not follow simple closure of the vessels by ligation.

The second item to be considered refers to the course of the bullet and the character of the damage done by it. Nothing can possibly be more uncertain and erratic than the track of the missile through the body. A contracting muscular fiber, an edge of fascia, the elasticity of the skin, a surface of bone, or a distended knuckle of intestine, each and all of these at times present obstructions sufficient to divert it from the direct line of its flight. It is certainly astonishing what very extensive and severe lacerations of the intestines are produced by so small a bullet as one of caliber No. 22, Fig. I. C.; the entire circumference of the bowel at some points being mangled beyond recognition; again, it is equally surprising how minute are the perforations made by the large No. 44, Fig. 2. As a rule, the larger the caliber of the bullet the larger the wound.

Figure I.

An estimate of the direction of transit, based upon the points of entrance and exit, is purely conjectural, and furnishes no standard whatever by which we may judge of any supposed injury to any organs known to lie in such course. In one experiment, the bullet made four openings through the abdominal walls, and did no damage other than contusion of two knuckles of the small intestine and gouging the serous membrane.

The animal had a remarkably deep furrow along the course of the "linea alba." The bullet entered the right side of the abmen obliquely, two inches from the mid-line, perforated its walls, and coursing to the left, furrowed the peritonæum in its passage; was evidently deflected outwards, immediately before reaching the linea alba, by a knuckle of intestine, which it contused slightly.

Here it made its first exit through the walls, passed to the left side of the mid-line, again perforated the abdominal walls, and, furrowing the peritonæum upon the left side, finally made its second exit through the abdominal walls three inches to the left of the linea alba. Near its place of final exit, a second knuckle of intestine was found badly contused. The contusion was so severe and extensive that it was thought best to resect a length of one inch. The animal recovered.

In a second instance, the bullet entered the cavity about two inches to the right of the linea alba, on a line with the umbilicus, with a direction upwards and to the left side. It made its exit nine inches to the left of the mid-line, and just at the lower edges of the last rib. On opening the abdomen the stomach was found greatly distended, entirely concealing the other viscera from view, and presented two large perforations in its walls about two inches apart, from which some blood, mucus, and food were found running into the peritoneal sac. The wound to the right, in the stomach walls, was the smaller, and situated directly opposite the entrance perforation in the abdominal wall, having the same direction. The wound to the left in the stomach walls (two inches to the left) was the larger, very ragged, and had evidently been made by the bullet deflected forward at its first entrance into the stomach. After leaving the stomach the bullet impinged upon the inside of the abdominal walls just to the left of the mid-line, and then, instead of perforating them at that point, was again deflected upwards and to the left, merely furrowing the peritonæum along the remainder of its course to the point of exit mentioned. The wounds of the stomach were inverted, as it were, into the cavity of that organ, by bringing its peritoneal surfaces surrounding the wounds in contact with each other by means of the continued catgut suture. The abdomen was carefully cleansed of blood, etc., and the wounds in the walls closed in the ordinary way. The animal speedily recovered from the injury, without any uncomfortable symptoms. During the recovery from the effects of the ether, the animal vomited considerable quantities of blood, giving an additional evidence of the perforation of the stomach.

There were two cases in which the bullets perforated the abdominal walls, and in their transit did no injuries to the viscera, in which the points of entrance and exit were five and six inches apart. In each instance the only damage done was a furrowing and laceration of the peritonæum along their entire courses, the blood from the track of injury falling into the abdominal cavity. In one experiment, the bullet failed to penetrate the abdominal walls and was subsequently dissected from between the muscles. On opening the cavity, quite a rent was found in the spleen opposite to the seat of external bullet wound, from which blood was freely flowing. There was neither abrasion nor perforation of the peritonæum. This case may suggest the probable cause of death in some fatal cases from non-perforating wounds. The laceration was evidently caused by concussion alone.

Other instances might be cited to illustrate the exceedingly great uncertainty as to the course taken by the bullet, and as to the organs probably impaired. They would also confirm the possibility of perforations of the walls without accompanying injury to the contents of the abdomen. Still, no instance was shown of failure to produce a wound thereof when the bullet's course lay among the intestines. Their safety followed deviation by glancing.

Figure 2.

The wounds of the intestines may be many in number and situated very near to each other (Fig. 3) so that one resection including all the openings will constitute the only operation that furnishes relief.

Again, the openings may be few in number and widely removed from each other; and if each wound is large, and the damage to the tube extensive, such as is usually produced by a 32, 38 or 44 caliber bullet, three or four resections are necessary. The latter are the most difficult cases to manage and most fatal in their results. The position of the points of entrance and exit of the bullet in the intestines is subject to immense variety, even in simple cases. It may involve only the top of a knuckle of intestine, merely opening the cavity thereof. The points may be so near each other that only a half inch or less of intestinal wall separates them from each other. (Fig. 1, a.) The bullet may merely cut off the mesenteric junction opening into the cavity more or less freely. The intestine is often perforated transversely near the middle, or longtitudinally; in the latter case the bullet, entering at one point, courses along in the cavity of the tube for some inches, and then makes its exit.

Figure 3.

All of these varieties depend upon the situation of the intestinal folds with reference to each other at the time of the transit of the bullet. One case showed 10 complete perforations in 18 inches of length of the ileum, Fig. 3.

Extravasation of the contents of the tube was present in every instance where there existed the slightest degree of perforation. These contents were forced out into the peritoneal cavity, or on to the surface of the intestines, if the wound was large, by the bullet itself, and the normal tonic contractions of the bowels; and, if small, perhaps by the latter alone. This facility of extravasation agrees with my experience in wounds of the intestine in the human being. I have personal knowledge of two instances in which the medium-sized aspirator needle was employed to relieve tympanitic distension of the tube with success so far as getting rid of the gas was concerned, and giving great temporary comfort to the patient. Death ensued from the disease. Post-mortem examination in each case demonstrated the presence of fæcal extravasation at the seat of the needle puncture. It would not be an arduous task to collate instances of this accident in the practice of others, where this plan has been adopted. It is difficult to understand how any other result could follow a perforation, if there be contents at the seat of the puncture, when we remember how strong and constant is the action of the circular muscular fiber. It is stated that the protrusion or eversion of the mucous coat, which ensues very rapidly after complete division of the walls, acts as an immediate stopper of wounds of small size, say one-eighth of an inch in diameter. This may be true in incised wounds, but it was not shown to exist in a single one of the several hundred perforations coming under my inspection as made by the bullet. The latter tears away and lacerates the parts through which it passes, and perhaps paralyzes the muscular fibers in its immediate neighborhood, but whatever the cause, there was no instance in which the eversion of the mucous membrane was sufficient to prevent extravasation.

Recognizing the very deleterious influence of this material upon the peritoneal membrane, this fact of the great certainty of extravasation adds another point to the argument in favor of abdominal section in these cases, as furnishing the only means by which this source of trouble can be absolutely eliminated.

As part of the extravasated material from the wounds of the intestine, it was an exceedingly common thing to find intestinal worms of all kinds, and in large numbers protruding from the rents or free in the serous cavity.

In the treatment adopted during these experimentations, it was found necessary to make an extensive external incision, freely exposing the abdominal cavity, in order that all the viscera might be thoroughly and carefully examined, and every wound brought within reach. In a majority of instances the median line gave space enough, in two the bleeding vessels could not be reached without a lateral prolongation toward the flanks.

There was no reason to suppose that the extent of the incision added very much, if at all, to the gravity of the operation. After opening the abdomen, the intestines were all turned out, critically examined for perforation or contusion, the situation of these fixed, and the hæmorrhage therefrom controlled by means of the snap forceps, after which wounds of special organs were sought for. If the substance of the spleen or the kidney was found perforated, the organ was immediately removed after ligating its blood-vessels, the stump being returned to the abdomen. If slight lacerations only at some point on the surface had been produced, these were closed by bringing peritoneal surfaces of the organ over the wound by means of the continued suture.

The peritoneal sac was then carefully and thoroughly cleared of blood and other extraneous substances by repeated sponging or irrigation. The intestines, which during this process had been protected by being enveloped in towels wrung out of warm water, were now cleanly sponged, while all unwounded portions were returned to the abdomen.

It seems to be of little consequence whether or not the intestines be returned to the cavity in any definite order — in fact, it is doubtful whether they are ever returned precisely to the same positions they originally occupied before being disarranged during the operation. Still, some care must be used in order to avoid the accident which happened in one experiment. After the divided ends of the intestine had been united, it was found that during the manipulation one of the ends had in some way been passed through an opening in the divided mesentery, so as to produce a figure of eight convolution in the tube. It was left in this shape. The animal recovered, and I have the specimen with me to demonstrate the perfectness and security of the union in the intestine at the place of reunion. The animal was sacrificed to secure the specimen six weeks after the operation. The abdominal cavity was quite free from evidences of inflammation, except where the misplaced folds lay in contact with each other. At this point slight peritoneal adhesion had formed between them.

Where several wounds occurred rather close together, severe enough to destroy a considerable portion of the integrity of the bowel, one resection was made to include all of them, even when the length of intestine removed measured ten inches or more. Where the points of injury were widely separated from each other and extensive damage done at each point, several resections of a length of the tube just sufficient to include the injured portions were made.

In the former case, in which several inches of the tube were taken away, the mesentery was ligated as close as practicable to the intestine (Fig. 7), in sections corresponding to the number of blood-vessels going through it to the resected portions. The mesentery was then divided close to the intestinal wall, and a "V" shaped portion of it removed. After this, the tube itself was divided, and the wounded portion removed. One artery, always needing ligation, was found in the divided ends at the point of junction of the mesentery with the intestine. Before the final division of the intestine, its contents were pushed back out of the way, compression exercised upon its walls by a pair of forceps or a temporary ligature, in order to prevent extravasation of its contents through the divided ends. The mark of constriction made by the forceps or ligature, used to close the lumen of the bowel, was to be plainly seen several days after the operation. The safest compression can be made by an assistant's fingers. Results soon demonstrated the paramount necessity of carefully selecting the place for final division of the intestine, in order to avoid sloughing of the edges approximated together, the results being best in those cases where the division was made close to the point at which any given mesenteric artery approached nearest to the intestine, as compared with those where the cut was made in the intervals between any two branches of these vessels, and this was seemingly dependent on the better supply of blood belonging to the former cases. Immediately after division of the intestine, there followed an instantaneous, regular and considerable contraction of the caliber of the tube (Fig. 4, a), close up to the divided edge, caused by the action of the circular muscular fiber. The diameter was often diminished more than half by this contraction. This persisted for a time, but was soon followed by an eversion of the mucous membrane, which rolled out and over the constricted portion in a remarkable manner. (See Fig. 4, a, b and c.)

Figure 4.

This protrusion of the mucous membrane forms a serious obstacle to easy and close approximation of the ends of the bowel in the ef-orts to bring them together by sutures; and, when turned into the bowel during such procedure, diminishes its caliber considerably, although it was not demonstrated that the obstruction was ever sufficient to prevent the passage of the intestinal contents. Several ef-orts were made to get rid of it, and overcome the seeming delay caused by its presence, but all these were finally abandoned.

It was pared away with the scissors; it was dissected up from the other coats for a quarter inch from the edges, but the conclusion was finally reached that instead of being a harm, its presence was useful in giving support, protection, and perhaps vascularity to the freshly sutured edges; at least, in all instances where it was removed, the stitches were found torn out and union defeated; in no instance where it was left entire did there fail to be union in some part, and no sutures gave way when properly applied.

Figure 5.

In all instances where a perforation was severe enough to require a resection of the wounded part, it was found advantageous to leave, if possible, a strip of the bowel near the mesenteric junction (Fig. 5, A), taking out the wounded portion by means of a "V"-shaped incision. The part left acted as a support to the wound, avoided division of the blood-vessels at this point, opposed the action of the longitudinal fibers, and in no instance in which this plan was adopted was there any appearance of separation of the wound or any displacement of stitches. In perforations through the stomach, the wound did well after drawing the peritoneal surfaces some distance from the edges thereof, over it by means of the continued suture, thus converting it into a linear wound (Fig. 6 A). The same plan was adopted with success in abrasion and small perforations in the small intestines. (Fig. 6 B.)

This way of treating the bullet openings in the bowel is susceptible of much wider application than would appear possible at the first glance. I am quite well satisfied that it will safely take the place of excision in not a few cases of quite severe injury. The torn edges of the wound can be turned in, and peritoneal surfaces fastened together, even in large wounds, with perfect confidence in the result of safe and secure adhesion following.

Figure 6.

It seems probable that by far the greater number of successful cases will follow a single resection, even if that include a number of perforations, and involves eight or ten inches of bowel, in comparison with those cases where several excisions are made of wounded portions widely separated.

Perforations passing through the mesenteric surface of the intestine were found the most difficult to treat, and even if slight seemed always to require a complete excision. A partial excision of this surface of the bowel resulted in an acute-angled elbow which never did well.

The point of attachment of the mesentery with the bowel will usually be found the most troublesome to manage, in applying the sutures in restoring a complete division. (Fig. 5, B.) It is quite difficult to so place the sutures as to secure a perfect reinversion of the mucous membrane, to bring serous surfaces fairly in contact with each other, and to get a sound junction. The difficulty arises apparently from the manner in which the folds of peritonæum separate from each other before passing on to invest the bowel, leaving a little triangular interval filled with loose connective tissues, fat and blood-vessels. Now, if the suture fails to include the muscular coats of the intestine as well as the peritonæum at this point, the junction will surely give way and extravasation result. To make this point secure, the greatest care must be taken in placing at least three sutures (Fig. 5, B), this number being usually quite enough to include the troublesome area, and these should always be the first sutures applied. In placing the remaining sutures to complete the junction after placing the three sutures mentioned, at the mesenteric surface, it assists materially in the ease of application, saves time, and especially avoids trouble from the everted mucous membrane, to apply one at the most convex surface, and then one half way down, on each lateral surface. After this is done, the remainder can be introduced easily and rapidly. If introduced in a regular series, one after the other, all the way around, it is a very slow process; the mucous membrane is always in the way, the needle openings in the intestines are apt to be uneven, and it is altogether the poorest plan of proceeding. The advantages mentioned as gained by taking the course suggested, are certainly all of them items of importance, and have some bearing on the result. At best, these procedures will be found very prolonged and tedious. The material used by me for sutures was silk and catgut—the latter for the continued, the former for the interrupted ligatures. No. 1 catgut; No. 2 silk. The needles were the full curved round needle, or ordinary straight sewing needle; the latter is the best. The sutures were introduced about the third of an inch, never less, from the divided edges, made to include the peritoneal and muscular coats, and brought out just free of the edge on one side, and were then reintroduced close to the edge, and made to include about the same amount and kind of tissue on the other side, being *very sure not* to allow the nedle to pass into the intestinal cavity. (Fig. 11.) Mr. Howse,* of London, proved conclusively in his cases of gastrotomy, that the fact of entrance of the needle into the cavity of the tube, carrying the thread with it, made the difference between success and failure, cases dying from peritonitis and extravasation when the entry occurred, and recovery following when the thread included only the peritonæum and muscular coats.

1. Peritoneum.

2. Muscular Coat.

3. Mucous Coat.

Figure 11.

Again, the everted tissue should be turned in before introducing the needle, so that it will pass through the rim of constriction. If entered too far away from the divided edge, too much tissue is turned into the intestine. When the mucous membrane was turned in, and the suture tightened, two broad surfaces of peritonæum were brought in contact. This you will recognize as Lembert's suture (Fig. 6, B), with one change. Lembert directs that only one and one-half line in width of tissue should be taken up by the suture. This amount of tis-

* Mr. Howse was the first surgeon to use the double row of sutures for the junction of serous surfaces together. Czerney's suture is an application of it to intestinal wounds. Its use is altogether too tedious, and gives no better result than the single suture including sufficient time.

sue will do very well in the closure of small slits, for which it was intended, and to which it was applied; but complete resection needs a much firmer hold to withstand the strain of peristaltic movements. *The fact is, that it makes no difference whatever what kind of suture is used, so that the principle of positively securing the application of two broad surfaces of peritonæum in contact with each other is certainly carried out. Jobert's, Gely's, and Czerney's double row of sutures were all given a fair trial*, but none of them resulted as well as this modified Lembert stitch. It never failed to be followed by good union when properly applied, with peritoneal surfaces brought together around the entire circumference of the intestine.

Figure 11.

The greatest number of mishaps followed drawing the sutures too tightly, which, if done, leads to death of the applied edges, and, of course, to failure. They must be drawn only sufficiently close to bring the surfaces fairly in contact, the subsequent swelling from obstructed circulation will hold the surfaces firmly together until glued to each other by the rapidly forming adhesive material.

The interval left by the incurving of the edges of the bowel, immediately after the completion of the operation, was found entirely obliterated, and the sutures covered up by effused lymph at the end of twenty-four hours. In one or two instances, where very small openings had been made in the bowel, they were occluded by passing a suture around the perforation, a short distance from its margin, pushing the wound into the cavity of the intestine, and then by tightening the suture the peritonæum was drawn together over it; a very satisfactory plan of procedure where circumstances will permit its application.

Figure 7.

The question of the proper disposition to be made of the divided mesentery, after removal of some length of intestine, is an important one to decide. No plan adopted proved entirely satisfactory. Previous to separation it was ligated in sections (see Fig. 7); the part beyond the ligature is apt to mortify and thus prove a focus for fatal inflammation. The tissue of the mesenteric membrane is not very vascular, and the vitality of the distal portion of the stump is seemingly best provided for by causing it to adhere to surrounding vascular parts.

In some cases the stumps were left free in the abdominal cavity; these all did badly, each showing mortification. In others the different sections were all included in one suture and then stitched to the bowel at the seat of operation, making as nearly as possible a continuous surface of mesentery.

These did much better, there being few instances of sloughing. When sloughing occurred, it seemed to be dependent upon and follow a too tightly fastened ligature. This method above mentioned of treating the divided mesentery is useful in another way: it gives support to the bowel at the point of resection, maintains the intestine in proper position by preventing bending, and also leaves fewer raw surfaces free in the serous sac. This last, a condition acknowledged to be the frequent source of serious trouble from faulty adhesions to surrounding organs, and from furnishing points from which septic absorption takes place.

A plan of dividing and treating the intestine and mesentery has been suggested* to me as a possible improvement on those already noticed. It is really an application of the plan already recommended in single perforations. (Fig. 5, A). This is to make the separation through the intestinal walls three-eighths of an inch on either side of the mesenteric attachment

Figure 8.

(Fig. 8), tear away the mucous lining of the retained strip of bowel (Fig. 9), and draw the peritoneal surfaces thereof together by the continued stitch. (Fig. 8). This would avoid division of the blood-vessels going to the bowel, do away with the necessity of using ligatures, and leave no raw surfaces free in the abdominal cavity. The opening formed by the folding together where the bowel-ends are united, should be closed by the continued suture. (Fig. 10). This method was adopted in one experiment with an excellent result.

Figure 9.

Figure 10.

Bleeding from slight lacerations of the spleen, kidney, or liver can be controlled by actual cautery lightly applied, perhaps the very best method to adopt. If the wound is a complete perforation of the body of the organ, the hæmorrhage is very great, rendering extirpation of the entire organ apparently the only sure way of surmounting the difficulty.

* Dr. John Bartlett, Chicago.

Quite frequently the entire mass of the greater omentum seemed to require removal. The bullet in the transit not only perforated it here and there, but passed along between its folds as well, leaving injured tissue and blood-clots of considerable size in its track. These clots disseminated themselves in the meshes in such a way as to entirely prevent their removal without tearing the tissue to shreds. When this condition was present in any degree the mass was amputated, after ligation, in sections. In a few instances these stumps gave rise to trouble, either from recurring hæmorrhage or mortification of the distal end.

In the aftertreatment it was often necessary to administer morphia to secure quiet. Very careful attention must be paid to the amount and kind of food given for some time after apparent recovery. One experiment resulted in failure after the lapse of three weeks from date of operation. The animal was lively, running about as freely as ever, all the functions normal, and the external wounds all healed, when it suddenly sickened and died, having tetanus accompanying rupture of the intestine, several inches above the seat of resection. Post-mortem examination showed masses of food and grit and greasy cloth, occluding the intestine, and distending it so enormously that rupture was produced; the tube at the seat of the operation was patulous and nearly of usual size. This animal was lost solely through neglect in the matter of feeding. Milk alone was given in all other cases for some weeks after operation. Certainly this is a matter of great importance, and suggestive of the proper care to be given after all such operations. Extreme emaciation occurs during the first week following the operation, and, if there is shown any likelihood of recovery, there follows a voracious appetite, which should be very sparingly gratified.

The circumstances under which these experiments were done, were such that it was absolutely impossible to carry out full antiseptic appliances. The external incision was treated with iodoform and oakum or absorbent cotton, and with two exceptions healed by first intention.

The bullet wounds through the abdominal walls were not probed nor disturbed in any way. Occasionally, when large and much contused, iodoform was poured on them. In only two instances did they suppurate or give rise to any trouble whatever, crusting over and healing rapidly. This result clearly enforces the rule of not disturbing the track of a bullet through the soft parts unless the most urgent reasons call for interference. The damage of a serious nature is not in the abdominal walls, but in the cavity; the nature of it can be better ascertained and the most satisfactory treatment adopted, after section through the linea alba, rather than by enlargement of the wound of exit or entrance, if any surgical interference be instituted.

In gun-shot wounds of any part of the body, it is not the injured muscular tissue or facia that causes grave concern, but the torn arterial trunk, or severed nerve, or fractured bone made by the missile, and here, too, incisions out of the course of the bullet track often furnish the best exposure of the parts for manipulation.

None of the wounds of entrance were perpendicular to the surface of the abdomen. All were more or less obliquely directed through the component tissues of the walls, so that they were valve-like in character and tended to close spontaneously. None of these cases presented any extravasation of the contents of the intestines through the external wounds, notwithstanding the lacerations of the tube were often very extensive, and considerable quantities of fæcal matter were found in the peritoneal sac. The conclusion naturally follows, that the discharge of such matters, through the external openings, is not of frequent occurrence after the wounds under consideration. The absence thereof is far from being proof of the non-occurrence of perforation of the intestine.

It can scarcely be expected that extravasation through the wounds in the abdomen will often happen as an immediate occurrence. This is most likely to occur, if present at all, several days after the injury, following adhesion of the bowel to surrounding parts, and the accumulation of considerable quantity of matter.

There is no reason to suppose that interference with the adhesions to be met with in operations, done some time after the injury, would be followed by any worse consequences than that which follows their disruption during the performance of operations for ovarian or other tumors. The hazard supposed to attend their severance is certainly exaggerated. With a clean cavity they will do equally well in all cases.

These experiments have not developed any data which will aid in the positive diagnosis of the severity, or extent, or kind of injury done to the viscera, or render such diagnosis less difficult than heretofore, previous to abdominal section.

They go a step in advance of this by supporting the assertion that it is absolutely useless to expect immunity from perforations of the intestines when the bullet has traversed the cavity. It seems, and is infinitely more reasonable to subject a patient to the slight risk of an abdominal section, showing unwounded intestines, than to allow him to pass through the fearfully deadly peril of wounded intestines unrelieved, on the barren supposition that they may have escaped injury.

Some uncertainty as to its necessity is likely to arise, except in those cases showing extravasation of the contents of the bowels, or those where the free loss of blood, as indicated by the usual symptoms accompanying such accident, calls for aid. When doubt exists, and a critical condition of the patient argues severity of lesion, abdominal section surely seems to promise relief that can come in no other way. Exploratory incision of the abdominal walls has been done so often, and with so little hazard, as to entitle it to be classed as a procedure in itself almost destitute of danger. Such a conclusion is certainly supported by the results developed during these trials. The rule was, no trouble whatsoever from this incision.

No deduction can more justly or positively follow, as the result of these experiments, than that an incision *de novo*, through the linea alba, is the best method of procedure in the treatment of the class of wounds under consideration; a plan far preferable to enlarging either of the openings made by the bullet. It at once gives command over the entire cavity; therefore any lesion likely to result in harm is far less liable to be overlooked; it is the least vascular part of the walls; incisions thereof are more easily and perfectly co-aptated than elsewhere, heal readily and soundly, and as a consequence, the oncoming cicatrix is less likely to be followed by ventral herniæ.

Thirty-nine (39) animals were used in these experiments, exclusive of those dying from the effects of the anæsthetic. Two of the thirty-nine were used to demonstrate the effects of closure of the main branches of the mesenteric artery upon the nutrition of the intestines. Of the remaining thirty-seven (37), three cases died immediately after the shot or from the effects of profuse hæmorrhage; one having a division of the aorta just below the mesenteric artery; the second had a large laceration of the kidney, with a wound of the renal artery; the third, a laceration of both kidney and spleen. One case, No. 4, had tetanus three weeks after operation, and is given a special position, simply owing to the presence of this condition as a complication in the case. The post-mortem examination, as already mentioned, developed other conditions which would have caused death, and which were no doubt the cause of the tetanic convulsions. Twelve of the remaining cases died inside of twenty-four hours, either from severe primary or recurring hæmorrhage, and the effects of the very extensive character of the wounds. Two out of this twelve (12) were cases requiring removal of the pregnant uterus, accompanied with many perforations of the bowel; death in both occurred from secondary hæmorrhage from uterine stumps — the ligature having slipped. Three (3) more had slight lacerations of the spleen and numerous perforations of the intestine. The spleen was removed and several inches of the tube excised in each case. In three (3) others, from twelve to twenty inches of the bowel was excised, and many arterial trunks severed. One of the twelve (12) had rapid mortification of five or six inches of the entire caliber of the bowel, apparently dependent upon the division of two large mesenteric arteries by the bullet, and also the resection of six inches of the intestine. The remaining three (3) of the number dying inside of twenty-four hours, are classified as having died of shock. On all of them the damage done by the missile was of excessive severity. The bullet was of large size (38 or 44 caliber), and the fire-arms possessing great penetrating and lacerating power. There was not manifested in any case any recognizable evidence of shock aside from that following great loss of blood. The transit of the bullet made no noticeable impression upon the pulse or respiration. In every instance where signs of severe prostration became manifest through change in respiration or weakening of pulse, there was found profuse hæmorrhage to account for such condition. I am inclined to infer that the cases are exceptional indeed, in which purely nervous shock will give rise to symptoms severe enough to mislead one to perform an unnecessary ventral section; rather, when severe constitutional manifestations follow the passage of a bullet through the abdominal cavity, good cause for them will be found, as soon as the cavity is opened, in wounded viscera or blood-vessels, and this course will often be the only possible way of either actually saving life or even prolonging it. None of these twelve cases could possibly have lived longer than twenty-four hours after the injury received. Most of them would have died much sooner without the control of hæmorrhage, alone made possible by the opening.

Two cases of the series were subjected to the expectant treatment. These cases were chosen because their injuries did not seem very severe; the hæmorrhage was not great, and the prostration not extreme. Both died; the first in one day; the other lived five days. Post-mortem examination showed extensive extravasation of the contents of the bowel and septic peritonitis.

In one case an attempt was made to establish an artificial anus. The wounded intestine was resected, and the ends fastened to the edges of the abdominal incision. The animal died of septic peritonitis in three days. This trial was made early in the experimentation, before any definite plan of procedure had been settled upon. This is the only experiment that has given rise to any regret, for I feel satisfied that, with a fair junction of the bowel and a clean abdomen, the animal would have been saved.

Eighteen of the thirty-seven (37) have thus far been accounted for; of the remaining nineteen (19), ten (10) died and nine (9) recovered.

The ten fatal cases lived from three days to three weeks. Peritonitis from one cause or another seemed to be the precursor of death. In six of them, mortification of the ligated stumps of the divided mesentery, together with mortification of the edges of the recently united bowel, were present. In the one that lived three weeks, death was the result of intestinal obstruction, caused by the adhesion of a fold of the intestine to the stump of mesentery left free in the cavity. An acute flexure was produced at the point, against which the contents of the bowel had accumulated in large quantity. A rupture was found above this mass, through which extravasation had taken place. The inflammation was so intense that everything was matted together, and the specimen so horribly offensive it could not be preserved. There was no separation at the point of operation on the bowel; it was thicker here than elsewhere; but full distension with water was allowed without leaking. All of these cases demonstrate conclusively the necessity of great care in the manner of dealing with the divided mesentery, and in the application of the sutures which bring the separated bowel-ends together. The remaining four furnished evidence of separation of the recently united parts of the intestine at the mesenteric junction. In all of them the thread failed to include the muscular and fibrous coat of the bowel, holding only the peritonæum. The result was extravasation, and death followed.

It may be a matter of surprise to you that the percentage of successful cases presented is so small—nine out of nineteen of those surviving over 24 hours—so few out of so many. To me, knowing well the extremely adverse circumstances under which these experiments were performed, it is a matter of astonishment to have so many recoveries included in so few cases. It is suggestive to remember that all the recoveries followed the use of the modified Lembert method of bringing the peritoneal surfaces together, while in many of the failures, trials were made of other methods. Full six weeks have gone since the last case followed by recovery was subjected to operation. The first favorable case was treated four months ago. None of the animals present evidence of being other than in their usual health. The longest resection of intestine among the recoveries measured over six (6) inches and included four (4) perforations.

It is scarcely possible to do work of any kind under more disadvantageous surroundings than accompanied the performance of these experiments. The operative work was carried on, and the animals kept in the prosector's room of a medical college during the winter season, in the midst of the odds and ends and bad hygienic conditions of such a place. No better accommodations could be secured. The labor has been purely one of experimental inquiry, and not a striving after recoveries, implying a choice selection of attending circumstances and special preparations to that end; therefore, I judge it proper and fair to claim the results as satisfactory. These results certainly indicate that a better showing is likely to follow where more satisfactory control can be had over both patients and surroundings than was present during these examinations.

They clearly demonstrate that a hopeful expectation of recovery may be entertained after operation, and suggest the nature of the injuries produced, what accident to avoid, and what treatment to adopt.

My confidence in coming before you with no better record is assured, when I remember that all of you are well aware of the great mortality of these injuries, under all circumstances. It must be large, surely, when Dr. Otis, in the surgical history of the war, says the authenticated cases of recovery can be counted on the fingers of one hand. It cannot be said that operative interference in these cases has as yet an established position. Still, perhaps Dr. J. Marion Sims looked with prophetic eyes upon the future, when he closed the article already referred to with the following words: "I have the deepest conviction that there is no more danger of a man's dying of a gunshot or other wound of the peritoneal cavity, properly treated, than there is of a woman's dying of an ovariotomy properly performed. Ovarian tumors were invariably fatal till McDowel demonstrated the manner of cure, which has now reached such perfection that we cure from 90 to 97 per cent. of all cases. And by the application of the same rules that guide us in ovariotomy to the treatment of shot wounds penetrating the abdominal cavity, there is every certainty of attaining the same success in these that we now boast of in ovariotomy."—*British Medical Journal*, March 4, 1882.

In a rather quaintly-written but richly-laden book on surgery, by Herr. L. Heister, Professor, etc., written in 1739, there occurs this passage:

"When the intestines are wounded but not let out of the abdomen, and therefore the wounds are out of reach, the surgeon can do nothing but keep a tent in the external wound, according to the rules laid down at chap. V, and after this bleed the patient if his strength will admit of it, advising him to rest, eat abstemiously, and to lie upon his belly; the rest is to be left to Divine Providence and the strength of his constitution. But the question may be asked here whether a surgeon may not very prudently, in this case, enlarge the wound of the abdomen, that he may be able to discover the injured intestine and treat it in a proper manner. Truly I can see no objection to this practice, especially if we consider that upon the neglect of it certain death will follow, and that we are encouraged to make trial of it by the successes of others. Sacherus, in Programmate Publico, Lipsiæ, ed. 1720, mentions a surgeon who performed this operation successfully."

A period of 100 years and more has rolled away since Dr. Heister published his belief and reported recovery, to the time when Dr. Sims expresses his convictions—over a century of doubts, timidity, uncertainty, and gloomy misgivings, lightened only occasionally by some bold and resolute assertions. The future asks for action, and it is not unreasonable to assert that careful trials will accomplish successful results.

Avoiding any spirit of dictation, it seems proper to tabulate the following conclusions as an outgrowth of the experiments:

First. Hæmorrhage following shot wounds of the abdomen and the intestines, is very often so severe that it cannot be safely controlled without abdominal section; it is *always* sufficient in amount to endanger life by secondary septic decomposition, which cannot be avoided in any other way than by the same treatment.

Second. Extravasations of the contents of the bowel after shot injuries thereof are as certain as the existence of the wound.

Third. No reliable inference as to the course of a bullet can be made from the position of the wounds of entrance and exit.

Fourth. The wounds of entrance and exit of the bullet *should not be disturbed* in any manner, except to control bleeding or remove foreign bodies when present. They need only to be covered by the general antiseptic dressing applied to the abdomen.

Fifth. Several perforations of the intestines close together require a single resection, including all the openings. Wounds destroying the mesenteric surface of the bowel always require resection.

Sixth. The best means of uniting the wounded intestine after resection is by the use of fine silk thread after Lembert's method. It must include at least one-third of an inch of bowel tissue, passing through only the peritoneal and muscular coats, never including the mucous coat. The everted mucous membrane must be carefully inverted, and needs no other treatment.

Seventh. Wounds of the stomach, small perforations, and abrasions of the intestine, can be safely trusted to the continued catgut suture.

Eighth. Every bleeding point must be ligated or cauterized, and especial care devoted to securing an absolutely clean cavity.

Ninth. The best method of treating the stumps of divided mesentery is to save the mesenteric surface of the bowel as above indicated.

Tenth. *Primary abdominal section* in the mid-line gives the best command over the damage done, and furnishes the most feasible opening through which the proper surgical treatment of such damage can be instituted. Further, its adoption adds but little, if anything, to the peril of the injury.

(*To be continued.*)

## Figures and Tables

**Figure 1. f1:**
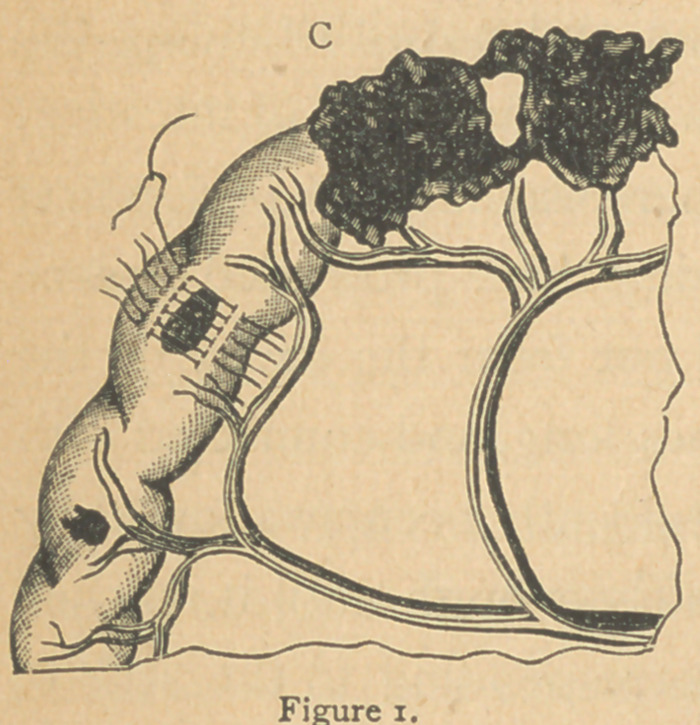


**Figure 2. f2:**
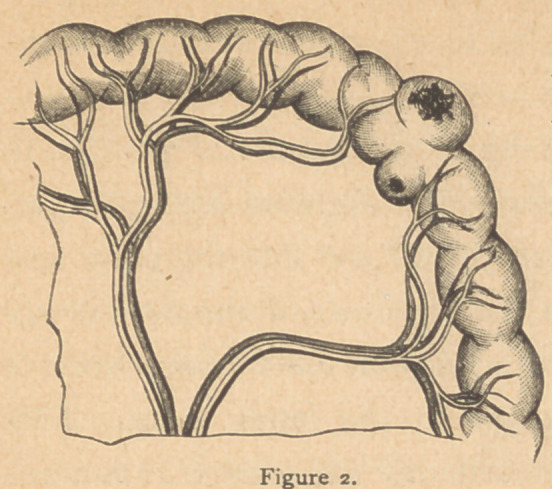


**Figure 3. f3:**
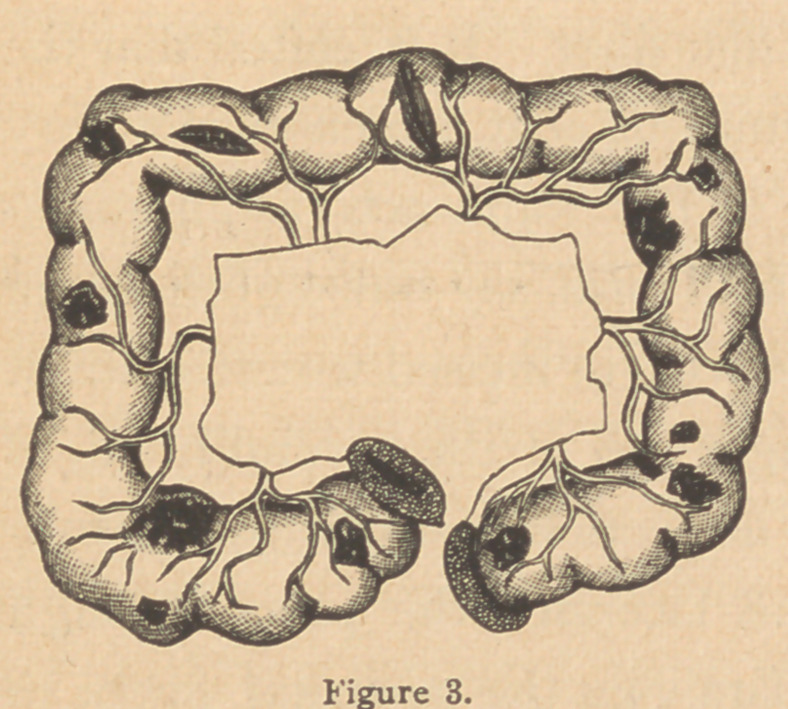


**Figure 4. f4:**
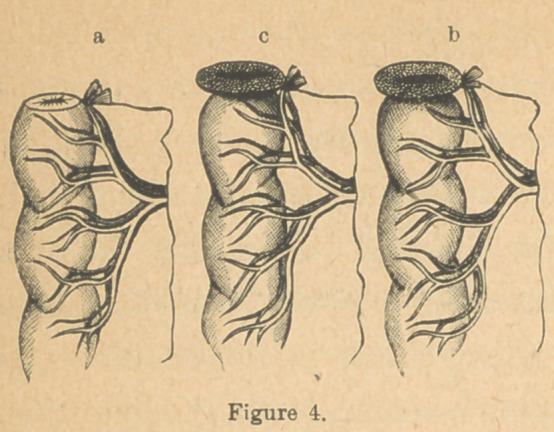


**Figure 5. f5:**
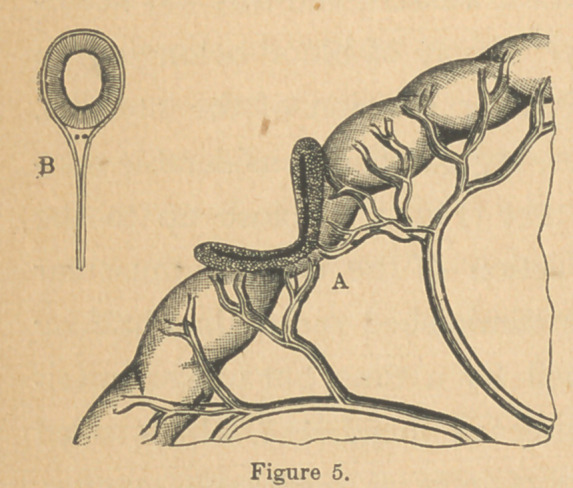


**Figure 6. f6:**
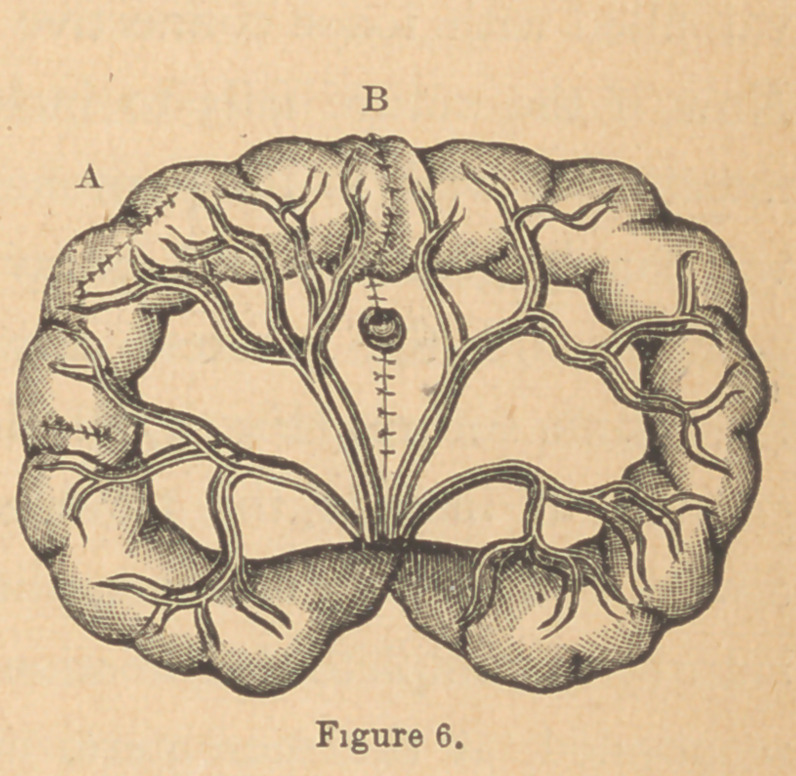


**Figure 11. f7:**
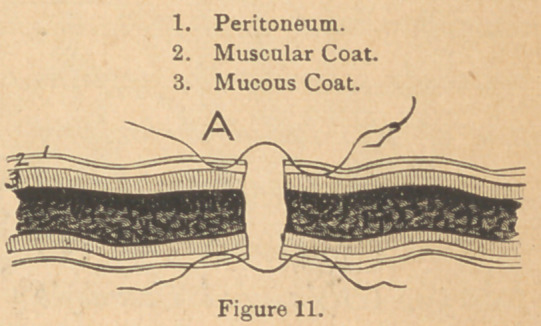


**Figure 11. f8:**
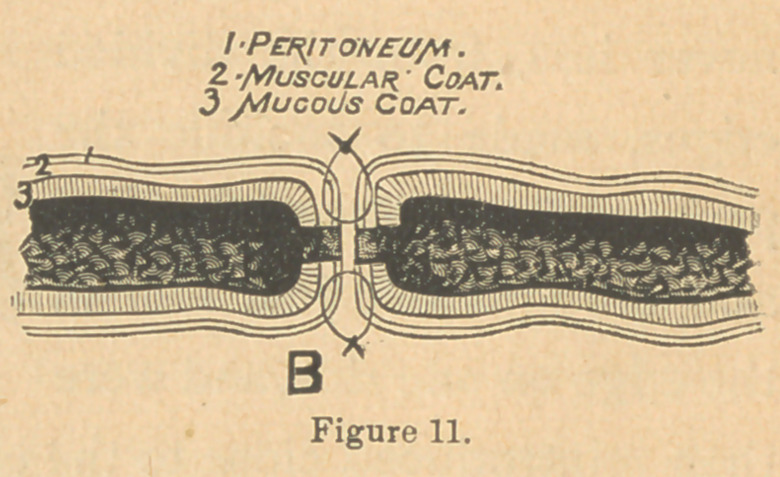


**Figure 7. f9:**
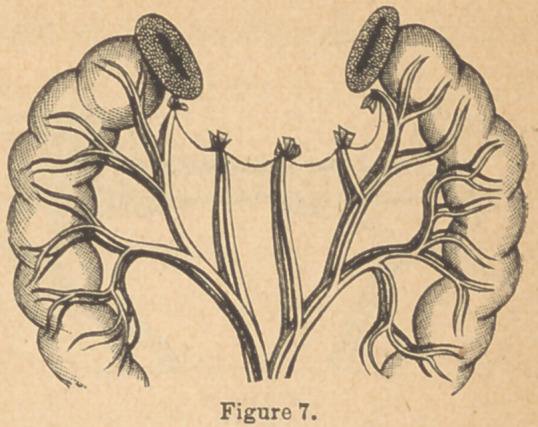


**Figure 8. f10:**
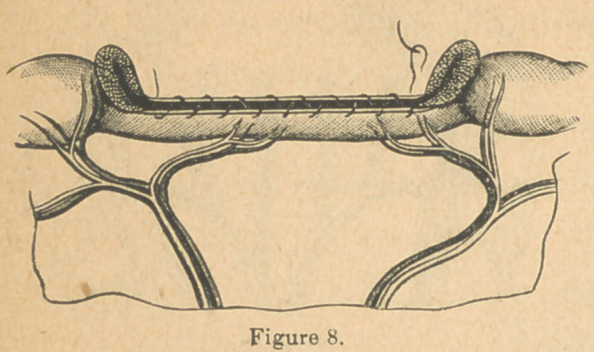


**Figure 9. f11:**
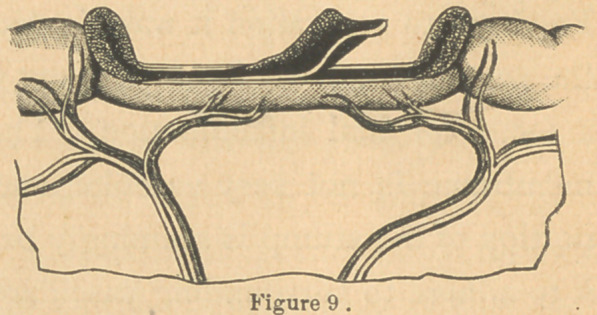


**Figure 10. f12:**